# Psychotherapists’ Ethical Dilemmas Regarding Online and Face-to-Face Psychotherapy During the COVID-19 Pandemic: Survey Study

**DOI:** 10.2196/69154

**Published:** 2025-09-17

**Authors:** Emilia Rutkowska, Joanna Furmańska, Cristiana C Marques, Maria João Martins, Håkan Lane, Johannes Meixner

**Affiliations:** 1 Department of Clinical Psychology and Psychoprophylaxis Institute of Psychology University of Szczecin, Poland Szczecin Poland; 2 University of Coimbra Health Services University of Coimbra, Coimbra, Portugalia Coimbra Portugal; 3 Johannes Gutenberg University Mainz Mainz Germany; 4 Department of Psychiatry and Psychotherapy, Brandenburg Medical School Immanuel Klinik Rüdersdorf Rüdersdorf Germany

**Keywords:** professional ethics, traditional therapy, remote therapy, epidemiological restrictions, international study

## Abstract

**Background:**

During the COVID-19 pandemic, mental health professionals were forced to find an appropriate way of working with patients that would ensure the continuity of therapy while considering the restrictions aimed at counteracting the spread of the virus. Online therapy has become an increasingly popular and common form of psychotherapeutic work. Emerging scientific studies have confirmed the positive effects of remote psychotherapeutic work. Nevertheless, modifying traditional and well-known forms of therapy or introducing completely new forms of remote therapy have been associated with several ethical concerns and challenges for psychotherapists.

**Objective:**

Due to the COVID-19 pandemic and the emerging epidemiological restrictions and recommendations, as well as new recommendations from psychotherapeutic associations, this study aimed to investigate the following: (1) Have psychotherapists experienced ethical dilemmas related to working online and face-to-face during the COVID-19 pandemic? (2) Was the occurrence of these dilemmas related to the therapists’ personal characteristics, such as age, sex, professional experience, or therapeutic approach? (3) What specific ethical dilemmas do psychotherapists point to in conducting online and face-to-face therapy during the COVID-19 pandemic?

**Methods:**

We conducted an international study with 177 psychotherapists from 4 European countries (Sweden, Poland, Germany, and Portugal) using a web-based survey. The psychotherapeutic approaches represented in the sample were cognitive-behavioral, integrative, psychodynamic-psychoanalytic, systemic, existential and gestalt, and Ericksonian therapy, among others. An interview comprising closed and open questions was used to collect data on psychotherapists’ personal characteristics, professional functioning, and ethical dilemmas encountered during online and face-to-face therapy.

**Results:**

Ethical dilemmas related to online therapy were reported by 58.7% (104/177) of therapists, while dilemmas related to face-to-face therapy were reported by 61% (108/177). The study showed that these dilemmas were independent of the personal and professional characteristics of therapists. Dilemmas related to online therapy were concern about online therapy, the issue of privacy and confidentiality of sessions, the effectiveness of online therapy, the issue of limitations that may hinder clinical work, and concerns related to the broader systemic and institutional context. In contrast, for the face-to-face form, ethical dilemmas mainly concerned health and safety, limitations in communication and quality of relationships due to wearing masks, and technical and logistical limitations.

**Conclusions:**

The issues considered here have not lost their relevance, because despite the lifting of restrictions related to the pandemic, some of the described dilemmas are similar to those related to coping with the risk and transmission of infection during face-to-face meetings. Moreover, the spread of online therapy means that the related ethical dilemmas are still relevant. The results indicate the direction of further consideration, the outcome of which should be specific ethical and legal guidelines that consider the concerns and dilemmas reported.

## Introduction

### Background

The first mention of the use of remote tools in psychotherapy can be found in the scientific literature in the 1950s [1]. Since then, along with the increasing computerization of everyday life, this type of solution has been increasingly used in psychotherapy. However, it was not until the announcement of the COVID-19 pandemic on March 11, 2020, by the World Health Organization [2] that psychotherapists worldwide had to adapt to the new reality of work caused by pandemic restrictions. Therefore, many psychotherapists who had never before offered psychotherapy remotely began to conduct sessions online. The growing popularity and prevalence of remote psychotherapy compared with the prepandemic period has also aroused the interest of many researchers (compared with the studies by Giordano et al [3], Rutkowska et al [4], and Furmańska et al [5]).

The results of the studies conducted showed that both therapists and patients had begun to look more favorably at this form of therapy [6-8]. The results of the analyses also seem promising. For example, the studies by Norwood et al [9], Bouchard et al [10], Probst et al [11], and Hanley and Wyatt [12] indicate positive effects of remote psychotherapy, while the studies by Chi et al [13], and Fernandez et al [14] believe that its effectiveness is comparable to that of face-to-face therapy.

Problems include limited observation of nonverbal communication, lack of access to certain therapeutic tools, and technical issues [15-17]. Many therapists report greater fatigue, feelings of less authenticity, and difficulty in building relationships with patients during online sessions [18,19].

Studies on ethical dilemmas in psychotherapy show that some dilemmas are specific to remote therapy, while others are characteristic of both remote and face-to-face therapy [20]. Stoll et al [15], in a review of 249 publications on the ethics of online psychotherapy, identified 24 arguments in favor of this form of work and 32 arguments against it. The most important arguments included increased access to therapy, flexibility, convenience, greater acceptance of this form of therapy, and economic benefits. Among the arguments *against* were issues related to privacy, confidentiality, and security; the need for special training of therapists; communication problems resulting from technology; and difficulties in managing emergencies. There was also the question of whether current ethical standards sufficiently considered the specificity of online psychotherapy [21]. Some codes of ethics contain guidance on what it means to work ethically remotely, but not all countries have supplemented them with this information (compared with the studies by Rutkowska et al [4] and Madej et al [22]).

Clinical experience shows that some people switched to remote work due to restrictions on social contacts and epidemiological recommendations, while others decided to continue face-to-face meetings in offices, which was also not free from ethical dilemmas and concerns about the risk of infection (compared with the study by Rutkowska et al [4]).

### Objective

Therefore, to identify the ethical dilemmas of psychotherapists and to better understand the differences in the perception of ethics in online and traditional psychotherapy, we decided to answer the following research questions: (1) Have psychotherapists experienced ethical dilemmas related to working online and face-to-face during the COVID-19 pandemic? (2) Was the occurrence of these dilemmas related to the therapists’ personal characteristics, such as age, sex, professional experience, or therapeutic approach? (3) What specific ethical dilemmas do psychotherapists point to in conducting online and face-to-face therapy during the COVID-19 pandemic?

## Methods

### Procedure, Participants, and Recruitment

The study was conducted in 4 European countries using a web-based survey: Sweden (northern Europe), Poland (eastern Europe), Germany (western Europe), and Portugal (southern Europe).

The following methods were used to assemble the research group: in the pilot phase of the study, short-term snowball sampling was used in the authors’ environment, and in the main phase of the study, purposeful sampling with elements of geographic stratification. (1) On the basis of official lists of members of various psychotherapeutic societies and associations in 4 countries, direct invitations to participate were sent; (2) in each state or province, requests for participation were sent to about 40 to 50 private practices and public institutions dealing with psychotherapy; and (3) recruitment advertisements were posted in specialized Facebook groups for psychotherapists. An invitation to participate was sent to each potential participant; a survey reminder was sent a maximum of 2 times. The combination of these methods resulted in a sample of 177 psychotherapists, diverse in terms of gender, age, experience, and therapeutic orientation [23,24].

After obtaining informed consent from the study participants, data were collected through a web-based survey in their native language. None of the participants received financial compensation for the study. The data collection in this study began in February 2022 and ended in March 2022.

The study participants were 177 psychotherapists from 4 European countries: Sweden (n=49), Poland (n=48), Germany (n=44), and Portugal (n=36). The average age of the surveyed psychotherapists was 47 (SD 13.8; range 23-80) years. Of the respondents, 28.2% (50/177 were men and 1.8% (3/177) not binary. The predominance of women among the respondents was consistent with statistics indicating that this profession is more often practiced by women [25,26]. The study group consisted mostly (123/177, 69.5%) of psychotherapists living and working in large cities. More than half worked in private practice (104/177, 58.7%) or worked both in private practice and public health care (63/177, 35.6%). The psychotherapeutic approaches represented in the sample were cognitive-behavioral (69/177, 39%), integrative (52/177, 29.4%), psychodynamic-psychoanalytic (28/177, 15.8%), systemic (11/177, 6.2%), existential and gestalt (5/177, 2.8%), Erickson therapy 2/177, 1.1%), and others (n/177, 5.6%). Most of the psychotherapists surveyed worked mainly with adult patients (10/177, 96%), followed by adolescents (aged 16-18 years; 67/177, 37.9%), and the smallest group of respondents with children (0-15 years; 28/177, 15.8%). On average, the respondents declared that they worked for an average of just over 5 hours per week as psychotherapists (0-40 hours; mean 5.4, SD 6.9 hours).

### Research Tools: Interview

For the purposes of the study, a interview questionnaire was created for this study consisting of closed and open questions. The closed questions related to the personal characteristics of psychotherapists (ie, age, gender, and nationality) and the characteristics of professional functioning (psychotherapeutic approach; professional experience: years of work; and experience with conducting online psychotherapy before the pandemic). The open questions were aimed at collecting data on ethical dilemmas arising in the professional work of psychotherapists and were formulated in the following way: (1) “Describe any ethical dilemmas you encountered while conducting online therapy, Please provide a specific example or describe how you dealt with this dilemma? What was the most difficult thing for you in this situation?” (2) “Describe any ethical dilemmas you experienced while providing face-to-face therapy during the pandemic? Please provide a specific example or describe how you dealt with this dilemma? What was the most difficult thing for you in this situation?”

### Statistical Analysis

Quantitative and qualitative analyses of the collected data were carried out to analyze the results and answer the research questions posed.

In this study, we used an explanatory sequential mixed methods design. In the first phase, a quantitative analysis was carried out (a closed-ended questionnaire: descriptive statistics, chi-square tests, and Mann-Whitney *U* tests), while the second phase involved a qualitative analysis (open-ended survey questions: subjected to thematic analysis according to Braun and Clarke [27]). This data sequence—labeled in the literature as QUAN→qual—first allows the scope and frequency of the phenomenon to be determined and then enables an in-depth analysis and the development of a classification of ethical dilemmas [28-30]. Statistical analysis was performed using the Jamovi program (version 2.3.28 [31].

The quantitative data analysis was conducted in several steps. First, the number and percentage of individuals who reported ethical dilemmas and those who did not were calculated for both the question regarding ethical dilemmas related to online psychotherapy and the question regarding ethical dilemmas related to conducting face-to-face sessions during the pandemic. Second, comparisons were made separately: (1) between the group of individuals reporting ethical dilemmas concerning online psychotherapy and those who did not report such dilemmas in this area and (2) between the group of individuals reporting ethical dilemmas related to conducting in-person sessions and those who did not report such dilemmas. These comparisons were made with respect to demographic variables related to the psychotherapist (gender and age) and variables related to their professional functioning (therapeutic approach; professional experience: years of practice; and conducting online psychotherapy before the pandemic). Comparisons for nominal variables were performed using the chi-square test, and for quantitative variables using the Mann-Whitney *U* test due to the violation of parametric test assumptions. Descriptive statistics for the individual dimensions were also presented.

Qualitative analysis was carried out in the form of thematic analysis, which allowed for the systematic identification and analysis of recurring themes occurring in qualitative datasets. The analysis of the collected data was conducted according to the methodology proposed by Braun and Clarke [27,32,33], in which the analysis process included 6 stages: becoming acquainted with the data, generating initial codes, searching for topics, reviewing topics, defining and naming topics, and developing a report. We adopted a constructionist epistemological perspective, assuming that psychotherapists’ statements co-create, rather than merely reflect, professional practices. Therefore, we were interested in latent (ie, hidden) patterns of meaning that extend beyond the semantic level. We conducted the qualitative analysis in the following steps in Textbox 1.

The coding process, conducted by 2 independent coders in accordance with the COREQ (Consolidated Criteria for Reporting Qualitative Studies) guidelines [34], produced a Cohen κ of 0.82 after the first round of coding all responses, indicating a satisfactory level of intercoder agreement.

**Six phases of thematic analysis applied in the present study (based on Braun and Clarke’s framework).**

- We began the analysis by carefully reviewing the data—they were read multiple times, which allowed for the initial identification of key ideas and directions of analysis.
- Then we generated the initial codes. We systematically encoded interesting features of the data in the entire set, which allowed us to focus on specific text fragments. These codes served as the starting point for further analysis and information synthesis. Coding was conducted primarily using an inductive approach.
- In the third stage, we consolidated and grouped the related codes into potential topics. Each topic was carefully analyzed in terms of its relevance to the main research question of our project.
- When reviewing the topics, we conducted a thorough check that the identified topics were consistent and adequately related to the analyzed data. This resulted in a thematic map that clearly outlined the structure and main themes of our analysis.
- Defining and naming the topics was the stage in which we precisely defined each topic and refined their names to clearly and precisely reflect the content of the analyzed data. Each topic is described, defined, and integrated into the narrative of the analysis.
- The last stage, preparation of the report, consisted of the synthesis and presentation of the results. We selected the most relevant pieces of data to illustrate the main themes of our analysis. The final report not only reported the results but also placed them in the context of the existing literature, highlighting their relevance and implications for further research in the field of psychology.

### Ethical Considerations

This study protocol was reviewed and approved by the ethical committee of Institute of Psychology of the University of Szczecin, approval number KB 18/2021. Written informed consent was obtained from participants to participate in the study.

## Results

### Overview

In accordance with the formulated research questions, to ensure the clarity of the presentation of the results, it was decided to focus first on presenting the answers to the research questions regarding online therapy—(1) Have psychotherapists experienced ethical dilemmas related to working online during the COVID-19 pandemic? (2) Was the occurrence of these dilemmas related to the therapist’s personal characteristics, such as age, sex, professional experience, or therapeutic approach? (3) What specific ethical dilemmas do psychotherapists point to in conducting online therapy during the COVID-19 pandemic?—and then for face-to-face therapy—(4) Have psychotherapists experienced ethical dilemmas related to working face-to-face during the COVID-19 pandemic? (5) Was the occurrence of these dilemmas related to the therapist’s personal characteristics, such as age, sex, professional experience, or therapeutic approach? (6) What specific ethical dilemmas do psychotherapists point to in conducting face-to-face therapy during the COVID-19 pandemic?

### Ethical Dilemmas Versus Lack of Ethical Dilemmas Related to Conducting Psychotherapy Remotely in a Group of Psychotherapists

In a survey conducted on 177 psychotherapists from 4 European countries, 58.7% (n=104) of respondents reported ethical dilemmas related to online work, while 41.24% (n=73) did not have such dilemmas.

By conducting statistical analyses, we checked whether the group of people reporting ethical dilemmas related to online psychotherapy differed from the group of people not reporting this type of dilemma in terms of variables related to the therapist (gender and age) and variables related to their professional functioning (psychotherapeutic approach, professional experience: number of years of work as a psychotherapist and experience in conducting online psychotherapy before the pandemic).

Statistical analysis showed no significant differences between the group of therapists who reported ethical dilemmas and those who did not in relation to gender (*χ*^2^_2.177_=2.1; *P*=.33), age of the psychotherapist (*U*=3505, *P*=.38), therapeutic approach (*χ*^2^_6_=11.2; *P*=.08), professional experience (*U*=3522; *P*=.41), or previous experience in online psychotherapy before the pandemic (χ^2^_1_=0.6; *P*=.42). These results show that none of the aforementioned factors significantly affected the occurrence of ethical dilemmas in psychotherapists in relation to remote work.

#### Ethical Dilemmas Related to Remote Psychotherapeutic Work

**Figure 1:**
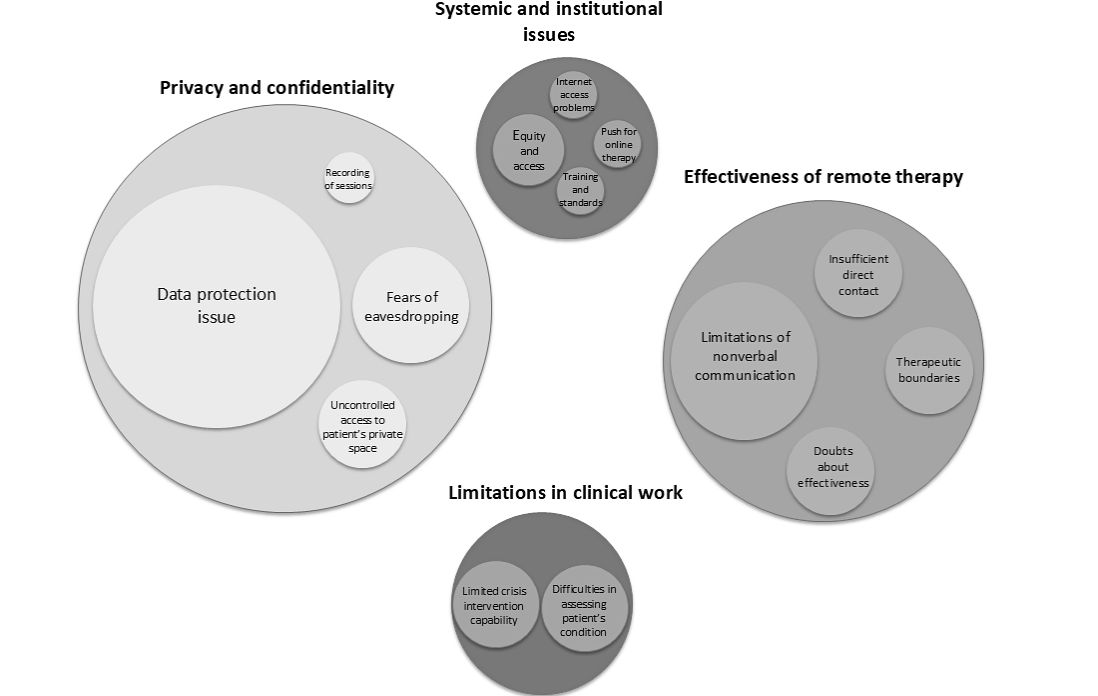
Ethical dilemmas related to online psychotherapy (chart 1).

The results of ethical dilemmas related to remote psychotherapeutic work are presented in Figure 1 and Table 1. Quantitative analysis of the obtained statements indicated that the minimum length of participants’ statements reporting ethical dilemmas regarding online psychotherapy was 1 word, and the maximum length of the statement was 68 words. Qualitative analysis revealed that these dilemmas can be classified into four main categories: (1) concerns about the privacy and confidentiality of remote sessions, (2) psychotherapists’ concerns about the effectiveness of online psychotherapy, (3) dilemmas related to the limitations inherent in online therapy that may hinder certain aspects of clinical work, and (4) concerns related to the broader systemic and institutional context and psychotherapeutic work in a remote form.

**Table 1:** Ethical dilemmas in the work of psychotherapists (N=104) related to conducting remote therapeutic sessions during the COVID-19 pandemic.

Themes, n (%)		Codes	Participants, n (%)
**Privacy and confidentiality, 53 (51)**			
	Data protection issue	Fear of data leakage; uncertainty about encryption; risk of unauthorized access	36 (34.6)
	Fears of eavesdropping	Possible eavesdropping; lack of environmental privacy	11 (10.6)
	Uncontrolled access to a patient’s private space	Exposure of home environment; limited privacy control	5 (4.8)
	Recording of sessions	Recording without consent; no control over recording	1 (1.0)
**Effectiveness of remote therapy, 30 (28.8)**			
	Insufficient direct contact	Lack of physical presence; weaker therapeutic alliance; limited online empathy	17 (16.3)
	Doubts about effectiveness	Skepticism toward the modality; comparative concerns about efficiency	7 (6.7)
	Limitations of nonverbal communication	Loss of body cues; absence of microexpressions	6 (5.8)
**Limitations in clinical work, 14 (13.5)**			
	Therapeutic boundaries	Blurred session framework; lower session intensity	8 (7.7)
	Limited crisis intervention capability	Difficulty in emergency response; no physical support	3 (2.9)
	Difficulties in assessing a patient’s condition	Diagnostic challenges; lack of environmental control	3 (2.9)
**Systemic and institutional issues, 5 (4.9)**			
	Equity and access	Digital exclusion; technological barriers	2 (1.9)
	Internet access problems	Poor connection; equipment deficiencies; no quiet space	1 (1.0)
	Push for online therapy	Economic pressure; commercialization of services	1 (1.0)
	Training and standards	Erosion of standards; training gaps	1 (1.0)

##### Privacy and Confidentiality

The first group of concerns was the patient’s privacy and confidentiality, which were feared by half of the psychotherapists reporting ethical dilemmas in general (53/104, 50.9%). The therapists emphasized both the anxiety related to limited control and reduced impact on ensuring the privacy of the session (uncontrolled access to the patient’s private space or fear of eavesdropping on the session) and concerns about technological data security (problems with data protection and concerns about possible recording of the session). In their statements, psychotherapists expressed concerns that the patient may not be in appropriate conditions to ensure privacy; for example, someone from the household or co-workers may hear (even unintentionally) the patient’s statements because online sessions took place in different conditions (office, car, kitchen, etc). The concerns expressed were also whether the patient was recording the session without the therapist’s consent and awareness. Some therapists were also concerned about whether the software they and their patients used was safe and resistant to cyberattacks.

##### Effectiveness of Remote Therapy

The second group of concerns was doubts about the effectiveness of psychotherapy conducted remotely, which was expressed by about 28.8% (30/104) of psychotherapists among those who reported ethical dilemmas in general. These doubts were expressed both in the form of general beliefs about online psychotherapy (7/104, 6.7% of psychotherapists expressed doubts about the effectiveness of online therapy) and resulted from the difficulties experienced by psychotherapists in working online. Therapists believed that remote psychotherapy was associated with insufficient direct contact, and there were clear limitations in nonverbal communication. For some psychotherapists, the lack of common physical space and contact was a substantially barrier. Therapists pointed out that they do not feel the emotions experienced by the patient in direct contact. Others pointed out that the screen does not allow for the reading of all nonverbal signals, which can make it difficult to understand the patient, impoverish understanding of the context of his or her speech, and thus hinder or reduce the effectiveness of proper diagnosis and treatment.

##### Limitations in Clinical Work

The third group of concerns of psychotherapists was related to the limitations in clinical practice (diagnosis and therapeutic interventions) that remote work with patients is burdened with, with 13.5% (14/104) of psychotherapists reported ethical dilemmas related to working online. Therapists reported limitations in the form of online sessions in situations that require crisis intervention, especially when it comes to threatening the life or health of patients. According to psychotherapists, the possibilities of remote work are limited (especially if someone lives, eg, in a different city or country from the psychotherapist, and an immediate psychiatric consultation or the intervention of additional services is required). Psychotherapists have also pointed out that it is difficult to have access to all the nonverbal symptoms of a patient’s behavior and sometimes to make a correct diagnosis via the internet. Some therapists have pointed out the inability to establish a deeper relationship with patients via the internet.

##### Systemic and Institutional Issues

Finally, the fourth group of psychotherapists’ concerns was related to systemic and institutional problems, which were reported by 4.9% (5/104) of the respondents. They signaled that psychotherapy is becoming less accessible to non–tech-savvy people; during the pandemic, some patients were unable to use psychotherapy due to restrictions and the need to work online. In the case of some people, this was related not only to the inability to access technology but also to the economic situation and lack of access to the internet. The lack of education in the field of online psychotherapy and the use of specialized programs through which online therapy sessions were conducted was also noted as an issue.

### Ethical Dilemmas Versus Lack of Ethical Dilemmas Related to Conducting Face-to-Face Psychotherapy in a Group of Psychotherapists

The second question concerned the ethical dilemmas associated with conducting face-to-face psychotherapy sessions during the COVID-19 pandemic. Ethical dilemmas in this area were reported by 61% (n=108) of respondents, while 39% (n=69) declared that there were no ethical dilemmas in this area.

The group of people who did not report such dilemmas was analyzed in terms of the gender and age of the psychotherapists, their therapeutic orientation, years of professional experience, and experience in conducting online psychotherapy before the pandemic.

Statistical analyses showed that sex (*χ*^2^_2_=3.2; *P*=.20), age of the psychotherapists (*U*=3185; *P*=.10), therapeutic approach (*χ*^2^_6_=10.5; *P*=.10), number of years of professional experience (*U*=3722; *P*=.99), and previous experience in conducting online psychotherapy before the pandemic (*χ*^2^_1_=0.001; *P*=.97) were not significantly related to the occurrence of ethical dilemmas related to face-to-face therapy during the COVID-19 pandemic. These results suggest that ethical dilemmas related to COVID-19 arise independently of the variables mentioned, indicating their universal nature among psychotherapists of different genders, experiences, and therapeutic approaches.

#### Ethical Dilemmas Related to Face-to-Face Psychotherapeutic Work During the COVID-19 Pandemic

**Figure 2:**
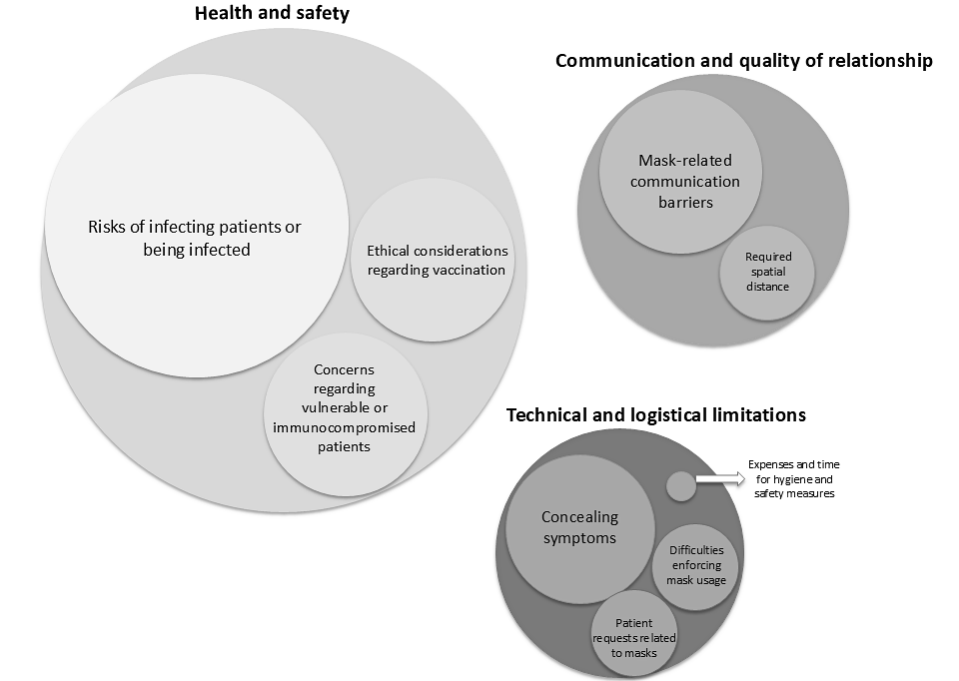
Ethical dilemmas related to face-to-face psychotherapy during the COVID-19 pandemic (chart 2).

The results of a detailed analysis of the statements of psychotherapists who reported ethical dilemmas related to face-to-face sessions during the pandemic are presented in Figure 2 and Table 2. Quantitative analysis of these statements showed that their lengths ranged from 1 to 59 words. Psychotherapists reporting ethical dilemmas related to the provision of face-to-face therapy during the COVID-19 pandemic expressed concerns about (1) the health and safety of face-to-face sessions, (2) limitations in communication and quality of relationships due to wearing masks during face-to-face sessions, and (3) technical and logistical limitations that can be a problem during sessions conducted directly in the psychotherapist’s office.

**Table 2:** Ethical dilemmas in the work of psychotherapists related to conducting face-to-face therapeutic sessions in relation to COVID-19 (N=108).

Themes, n (%)			Codes		Participants, n (%)
**Health and safety, 64 (59)**					
	Risks of infecting patients or being infected	Fear of infecting others; fear of contracting infection; perceived contagion risk		52 (48.1)	
	Ethical considerations regarding vaccination	Ethical dilemma about vaccination; respecting autonomy vs safety; vaccine-related boundaries		7 (6.5)	
	Concerns regarding susceptible or immunocompromised patients	Protection of high-risk patients; need safe solution; discussing risks with susceptible patients		5 (4.6)	
**Communication and quality of relationship, 20 (18.52)**					
	Mask-related communication barriers	Facial expression hidden; reduced nonverbal cues; muffled speech		18 (16.7)	
	Required spatial distance	Physical distance challenges; hard-to-read emotions; reduced safety or comfort		2 (1.8)	
**Technical and logistical limitations, 14 (12.9)**					
	Patient requests related to masks	Requests to remove mask; negotiating mask use		3 (2.8)	
	Expenses and time for hygiene and safety measures	Time‑consuming disinfection; additional costs of personal protective equipment		1 (0.9)	
	Difficulties enforcing mask usage	Reminding mask compliance; enforcement fatigue		4 (3.7)	
	Concealing symptoms	Patients hiding symptoms; breaking isolation guidelines		6 (5.5)	

##### Health and Safety

The first group of ethical dilemmas reported by psychotherapists regarding face-to-face sessions during the COVID-19 pandemic were related to health and safety; such dilemmas were observed in 59% (64/108) of the psychotherapists surveyed. Psychotherapists indicated that they were afraid of potential infection from patients, especially in the context of not knowing whether the patient was vaccinated and admitted that the need to ask for the results of COVID-19 tests was embarrassing for them. Psychotherapists were also concerned that by having contact with different people during psychotherapy sessions, they could also pose a threat to their own patients and expressed concern that they could infect others, for example, patients with autoimmune diseases who could not be vaccinated.

##### Communication and Quality of Relationship

The second group of concerns related to communication limitations and the quality of relationships related to (1) wearing masks during face-to-face sessions and (2) requiring physical distancing during the session. Dilemmas of this type were reported by 18.5% (20/108) of psychotherapists. According to psychotherapists, wearing masks during therapy sessions made full facial expression difficult for both patients and therapists as it hindered the perception of emotions and intentions by both parties. In addition, some therapists pointed out that because of pandemic recommendations, they had to maintain physical distance during sessions with patients to minimize the risk of transmission of infection, which, according to some, affected the comfort and quality of communication between therapists and patients. According to therapists, being forced to keep distance can be a barrier to establishing a fuller therapeutic relationship, affecting the sense of security and closeness, which are important in the therapy process.

##### Technical and Logistical Limitations

The third group concerned dilemmas related to technical and logistical aspects, which were expressed by 12.9% (14/108) of psychotherapists reporting ethical dilemmas related to conducting face-to-face sessions during the pandemic. Therapists emphasized that additional procedures related to the disinfection of surfaces and rooms were associated with a financial and time burden, which was another challenge in therapeutic work. There were also dilemmas among therapists regarding enforcing or withdrawing from mask requirements during sessions. They also indicated that they did not always know what to do when patients asked if they could take off their masks during the session, as they were afraid that this topic could lead to additional tension and affect the course of the session. Therapists also said that they did not know what to do in a situation where some patients coming to the office might hide the symptoms of the disease or did not comply with mandatory isolation.

## Discussion

### Principal Findings

**Figure 3:**
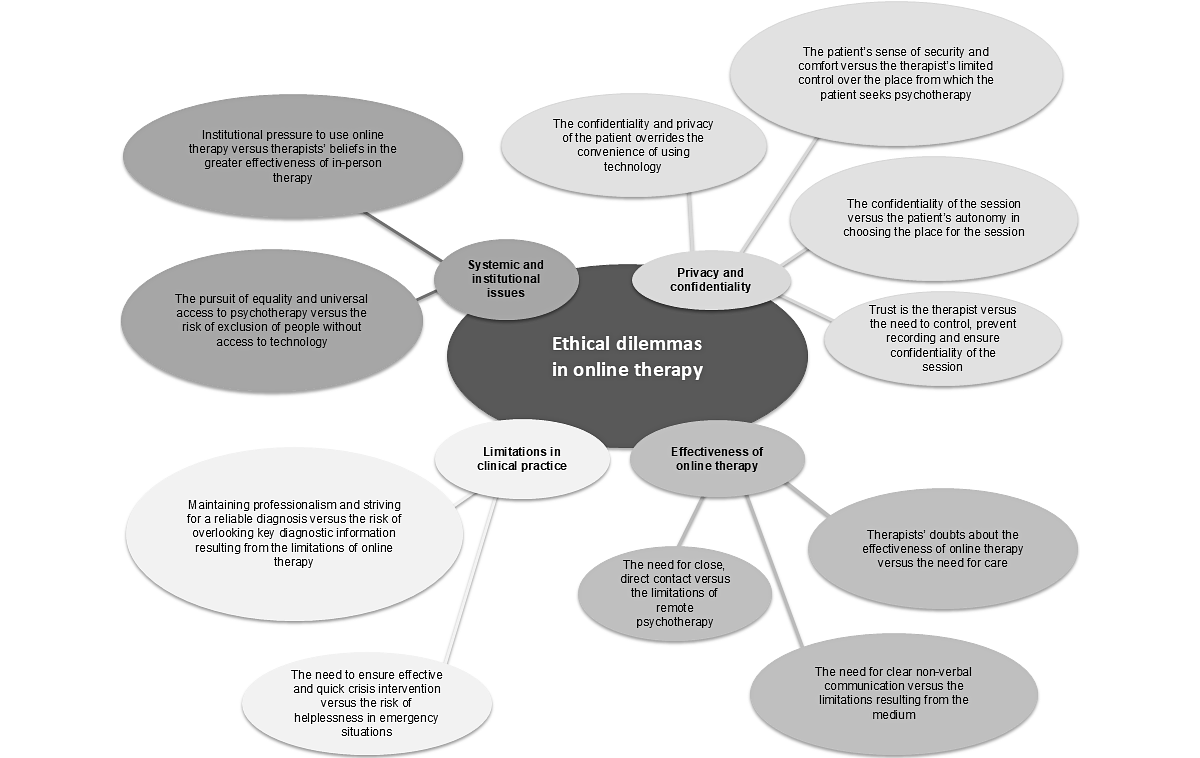
Ethical dilemmas in online psychotherapy (scheme 1).

**Figure 4:**
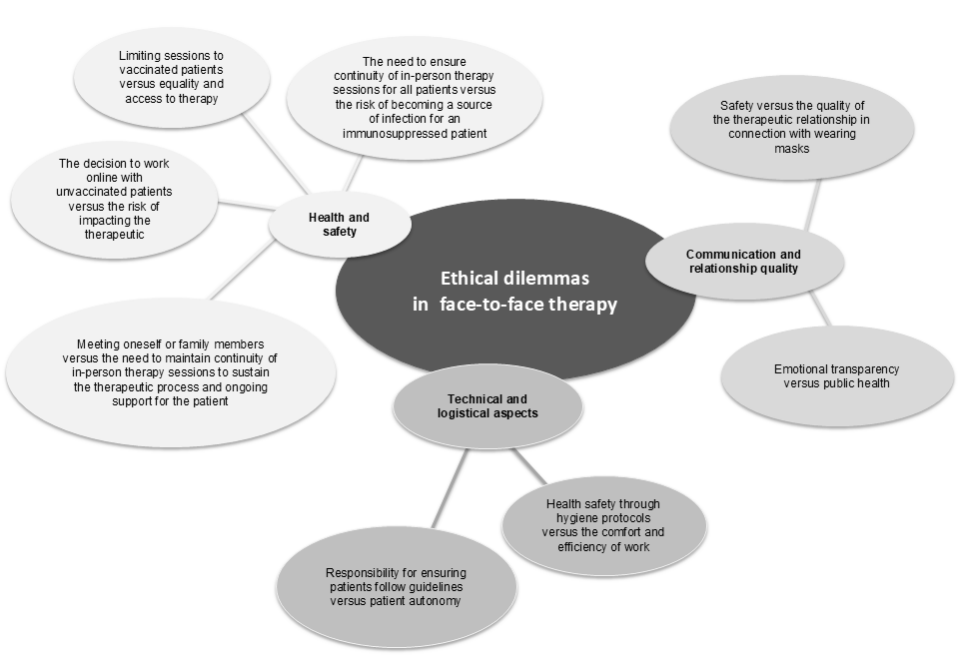
Ethical dilemmas in face-to-face psychotherapy (scheme 2).

Our study revealed the ethical dilemmas faced by psychotherapists; these dilemmas concerned various aspects depending on the form of therapy and were reported by a significant number of therapists (online: 104/177, 58.7%; face-to-face: 108/177, 61%). These results indicate the need for further research and development of standards and guidelines that could support psychotherapists in the delivery of their work in both forms of therapy. The ethical dilemmas identified in the study can be explained and interpreted using the presented Figures 3 and 4.

### Ethical Dilemmas Related to Online Psychotherapy

The study showed that 58.7% (104/177) of the psychotherapists surveyed had ethical dilemmas related to online psychotherapy during the pandemic and that these therapists did not differ in terms of age, gender, therapeutic approach they practiced, years of experience in the profession, or in terms of experience in conducting online psychotherapy before the COVID-19 pandemic from psychotherapists who did not declare ethical dilemmas.

Thus, the results of the study indicate the prevalence of ethical dilemmas related to online psychotherapy, regardless of age, sex, therapeutic approach, professional experience, or previous practice of online therapy. This suggests that ethical challenges in online therapy may be universal and common, resulting from the specificity of this form of work (compared with the studies by Avasthi et al [35] and Palomares and Miller [36]) rather than the individual characteristics of the therapist or the specificity of the therapeutic trend in which the therapist works. In addition, the increased popularity of this form of work in connection with the COVID-19 pandemic, as well as the lack or small number of precise recommendations on the ethics of online psychotherapy issued by professional societies, meant that many psychotherapists faced similar ethical dilemmas and the need to adapt to new professional realities. Perhaps it should be understood that ethical dilemmas and the challenges they entailed resulted from the need to adapt to new professional realities rather than from a lack of competence or experience. The obtained results and hypothetical explanations should be treated with caution; however, due to the relatively small research sample, which is not representative in terms of gender or place of work (dominance of women, and people working in large cities and private practices), the results merit further study.

### Categories of Ethical Dilemmas Related to Online Psychotherapy

A detailed qualitative analysis of the answers obtained allowed us to distinguish 4 main categories of ethical dilemmas related to remote conducting psychotherapy: privacy and confidentiality, effectiveness of remote therapy, limitations in clinical work, and systemic and institutional issues.

#### Privacy and Confidentiality

Psychotherapists faced dilemmas related to the conflict between (1) the confidentiality and privacy of the patient and the convenience of using technology; (2) the patient’s sense of security and comfort, and the therapist’s limited control over the place from which the patient uses psychotherapy; (3) the confidentiality of the session and the patient’s autonomy in choosing the place for the session; and (4) the conflict between trust in the patient and the need to control and ensure full confidentiality of the session, where the therapist, to believe in the patient’s honesty, may avoid suspicion, but the lack of control over the situation raises concerns about unwanted recording, which may affect the privacy and confidentiality of the conversation.

The results of this research are consistent with the analyses conducted before the pandemic (compared with the studies by Stoll et al [15] and Donner et al [37]), which indicate that the risks associated with confidentiality and privacy are among the most discussed disadvantages of using remote psychotherapy.

Without privacy and confidentiality, there is a risk that the therapy may not be fully effective [38] only because of the fear of opening up and revealing personal content, which could result from the fear that information from the conversation could be disclosed and overheard by third parties. Consequently, patients could refrain from delving deeper into private topics during online sessions, and low openness during sessions could reduce the effectiveness of psychotherapy.

In our research, therapists pointed to 2 potential sources of violation of privacy and confidentiality of sessions: technological and those resulting directly from patients’ behavior.

Regarding the technological invasion of privacy, a detailed specification of the sources of such threats can be found in the literature. Warnecke [38] lists the main causes of network security problems: electronic intrusion by a third party, online intrusion by an electronic service provider, and electronic surveillance by national security services.

On the other hand, when it comes to the invasion of the privacy of the session, the source of which may be the patient themself, it is often associated with the choice of a proper, private, and safe place, without the presence or possibility of intrusion by third parties, such as family members and roommates [39-43].

Interestingly, in this study, the therapists did not indicate themselves as the source of the breach of privacy and confidentiality during the session. However, it may also happen that it is the psychotherapist who does not take care of securing the space in which they work, making it vulnerable to accidental eavesdropping, or who use unsecured devices or software that does not meet security standards. It seems that this could have been due, for example, to the reluctance to admit mistakes, which could affect their professional image, or to the belief that technological security rests solely with service providers, which means that they do not see themselves as potential sources of breach.

In conclusion, we can say that regardless of the source of the breach of privacy and confidentiality, this is a key issue that psychotherapists using remote forms of therapy should take into account when planning to use this form of psychotherapy [44]. In addition, it should be remembered that to effectively counteract the abovementioned problems, it is important to implement guidelines and best practices that can minimize technological risks and educate patients on how to prepare the appropriate environment for the therapeutic session. These activities not only improve ethical standards but also support the creation of safer and more comfortable conditions for remote therapeutic work.

#### Effectiveness of Remote Therapy

The aforementioned conflicts focused on dilemmas related to (1) the conflict between the need for close, direct contact and the limitations of remote psychotherapy; (2) therapists’ doubts about the effectiveness of online therapy and the need to ensure continuity of care; and (3) the need for clear nonverbal communication and the limitations resulting from the medium.

Some respondents were not entirely convinced that online psychotherapy was as effective as face-to-face psychotherapy. Interestingly, this concern contradicts the results of scientific research, which show that both forms of psychotherapy are equally effective [13-15,45-47]. The lack of conviction by some therapists regarding the effectiveness of online therapy, despite the results of research indicating its comparability with face-to-face therapy, may result from little experience in conducting this form of psychotherapy (for a large number of psychotherapists, it is a new form of work) and the lack of observation of its long-term effects on their patients. It may also stem from a lack of skills in using remote tools. In addition, some specialists may believe that online therapy violates a certain intimacy of the relationship between the patient and therapist, introducing an element of the third party, ie, technology, while weakening the sense of closeness and security, which are crucial for an effective therapeutic process (compared with studies by Furmańska et al [5] and Bauman and Rivers [48]). The identified discrepancy between some therapists’ skepticism toward online therapy and its documented effectiveness can be interpreted in light of the technology acceptance model [49], which suggests that the intention to use technology depends not only on its perceived effectiveness but also on the subjective assessment of its ease of use. In this context, negative attitudes may reflect limited digital proficiency, concerns about the quality of the therapeutic relationship in the online environment, and a general resistance to changing clinical practice [50].

Beg et al [51] conducted a narrative review examining the literature on the use of artificial intelligence (AI) in psychotherapy from 2009 to 2023. Their results suggest that AI can enhance some psychotherapeutic interventions, but ethical integration of AI requires addressing issues related to privacy, trust, and interactions between humans and AI. Furthermore, from a research perspective, adherence to established reporting standards can protect academic integrity and enhance trust in technological progress [52].

An additional aspect that psychotherapists pointed out in our study, which was associated with doubts about the effectiveness of online psychotherapy, was the lack of physical contact and a shared physical space. According to the psychotherapists surveyed, this constituted a significant barrier to effective communication. It seems that this may also apply to difficulties in reading subtle emotional expressions and additionally refers to the fact that physical space plays the role of a carrier of emotions between people. In addition, it may make it difficult to understand the patient and consequently reduce the effectiveness of proper diagnosis and treatment (compared with the studies by Harris and Birnbaum [53] and Ford [54]), as well as hinder the identification of nonverbal signals indicating crisis situations, including threats to health or life, such as the presence of suicidal thoughts or tendencies [55]. At the same time, psychotherapists should be encouraged to base their clinical work on scientific evidence that supports the effectiveness of this therapeutic form, and to continuously monitor their own emotions, dilemmas, and attitudes toward online therapy, including, how these influence their evaluation of it.

#### Limitations in Clinical Work

Dilemmas from this group may be expressed in the form of conflicts between (1) the need to ensure effective and quick crisis intervention and the risk of helplessness in emergency situations and (2) maintaining professionalism and striving for a reliable diagnosis and the risk of overlooking key diagnostic information resulting from the limitations of online therapy.

The clinical experience of many psychotherapists shows that one of the significant limitations of online psychotherapy is the lack of a full physical presence, and difficulties in assessing nonverbal communication [8,15]. General appearance and behavior, affect, eye contact, and psychomotor functioning are important elements of mental state screening [56]. In psychotherapy, it is important for the therapist to notice the client’s emotions, understand them, and react appropriately. Sometimes, this can be difficult in remote psychotherapy because therapists often do not have access to proxemics as they may only see the client’s face. In addition, facial expressions are not always fully available because the face is not always seen accurately, due to factors such as lighting or screen quality. Hence, therapists may be concerned that they will miss something important during a session if it is held remotely [48]. In addition, among the dilemmas concerning limitations in clinical practice, the therapists surveyed drew attention to the problem of establishing a deeper psychotherapeutic relationship. It is worth noting that, although research shows that the effectiveness of online and face-to-face therapy is similar, when it comes to the psychotherapeutic relationship, the results of studies are sometimes inconclusive. Some of them show that the therapeutic alliance and the strength of relationships in online therapy are similar [9,57], while others indicate that psychotherapists note some differences in the therapeutic relationship in online and face-to-face sessions [48], especially in terms of the applicability of certain techniques in virtual reality [58,59], or the possible influence of the strengthening of the therapeutic alliance [60].

Therefore, psychotherapists may feel that diagnostically, the limited ability to observe subtle changes in the patient’s appearance, facial expressions, and gestures, may carry the risk of not noticing important signals, which may lead to an incomplete assessment of the patient’s mental state and sometimes misdiagnosis. Consequently, it is advisable to establish a procedure in a crisis situation at the level of the contract with the patient to provide the patient with contact details to local crisis support centers.

#### Systemic and Institutional Issues

Psychotherapists’ ethical dilemmas in this area included conflicts between (1) the pursuit of equality and universal access to psychotherapy and the risk of exclusion of people without access to technology and (2) institutional pressure to use online therapy and therapists’ beliefs in the greater effectiveness of in-person therapy.

When using media as a standard form of work, it should be borne in mind that both the psychotherapist and the patient must have digital skills and at least a minimum level of technological competence [21], as well as access to appropriate hardware or software. During the pandemic, owing to the restrictions introduced and recommendations of therapeutic societies (compared with the study by Lin et al [61]), which were mostly open to this form of work, therapists and clients faced the need to quickly adapt to the remote form of therapy, but they had to face related technological challenges on their own.

Therefore, it is advisable for psychotherapy institutions and associations to actively support the development of digital competencies among both psychotherapists and patients, offering dedicated training or conducting educational campaigns.

### Ethical Dilemmas Related to Face-to-Face Psychotherapy

Our study on ethical dilemmas in online and face-to-face psychotherapy in the era of the pandemic showed that 58.7% (104/177) of respondents had ethical dilemmas regarding online psychotherapy, and sessions conducted in a face-to-face format raised ethical dilemmas in 61% (108/177) of respondents. A comparison of these results indicated that the COVID-19 pandemic was a source of significant ethical dilemmas for therapists, regardless of the form of therapy, but the proportions were similar. The emerging dissonance between recommendations to limit social contact and the need to continue psychotherapeutic help may have exacerbated difficulties in deciding on the optimal way to work, especially for people who are less experienced in conducting online therapy or are skeptical about its effectiveness. In addition, the presence of dilemmas in both face-to-face and online psychotherapy during the pandemic did not depend on sociodemographic and professional variables but was rather an element of the specificity of this form of work in a pandemic situation. This situation was new to all therapists, which may explain why it was a common experience for all psychotherapists.

### Categories of Ethical Dilemmas Related to Face-to-Face Psychotherapy

The ethical dilemmas identified in the study are presented in Figure 2.

#### Health and Safety

The first group of ethical dilemmas regarding the conduct of face-to-face therapy in the era of the pandemic was related to concerns about their own and their patients’ health and safety. Psychotherapists were afraid that they would infect their families themselves, or alternatively, that they could transmit the infection to patients. They distinguished a group of immunocompromised patients for whom coronavirus infection can have serious health consequences.

Perhaps this dilemma prompted psychotherapists to make remote contact with patients more often. In the context of these challenges, factors such as experience in online therapy, physical distancing during sessions, and fatigue due to pandemic restrictions have led therapists to view the benefits of the remote form as an alternative to continuing to work in safe and comfortable conditions [4]. In addition, it should be noted that the results of the study suggest that therapists who showed a higher level of fear of contracting the coronavirus and the occurrence of health complications were more likely to see the positive aspects of remote therapy (compared with the study by Rutkowska et al [4]), while specialists who had direct contact with patients with COVID-19 presented higher levels of anxiety and stress than specialists who did not have such contact [62].

Although fear of infection may be one of the reasons why therapists either decided to have remote sessions or strictly followed all pandemic recommendations, it is worth remembering an additional factor. It can be assumed that psychotherapists experiencing this fatigue may lose the motivation to consistently follow recommendations, which potentially leads to the activation of defense mechanisms that minimize the perceived threat. Pandemic fatigue manifests itself in a reduced motivation to take protective measures, comply with recommendations, and withdrawal from seeking information about the pandemic [63-65].

In addition, working with unvaccinated patients during face-to-face sessions raises doubts. The results obtained and clinical experience show that psychotherapists faced ethical dilemmas when working with unvaccinated patients, resulting from the need to reconcile the values of health, safety, and respect for patient autonomy. On one hand, psychotherapists felt that they had a duty to ensure safe working conditions for both themselves and their patients, but in a situation where the patient is unvaccinated, they may be afraid of the risk of infection in in-person therapy, which could be related to their fear of their own health and that of other patients. On the other hand, each patient has the right to make their own decisions, including decisions about vaccinations. Therefore, while respecting the patient’s autonomy and choice, therapists may experience internal conflicts with their own views if they disagree with the patient’s attitude or see potential health risks resulting from the lack of vaccination. It is worth noting that during the pandemic, moral dilemmas related to health care have arisen concerning whether physicians and other health care providers should treat patients who have declined the COVID-19 vaccination [66]. Therefore, we also have the dilemma of whether limiting in-person sessions only to vaccinated patients would not violate the principle of equality. In such cases, are they not excluding unvaccinated patients from access to therapy? On the one hand, they want to provide safe conditions, but on the other hand, they must take care of equality in access to health services. In addition, it should be noted that the decision to work exclusively online with unvaccinated patients may affect the therapeutic relationship and cause a sense of rejection or judgment from the patient. At the same time, it seems that online work may not always be as effective as in-person therapy, especially for patients who prefer face-to-face contact.

Transparent communication with the patient, including explaining to the patient the limitations of in-person sessions for unvaccinated patients and emphasizing concern for the health and safety of all parties may be helpful in solving this dilemma. Therapists may also consider introducing educational elements to improve patients’ understanding of health and safety practices. In conclusion, the ethical dilemmas of working with unvaccinated patients are a complex issue in which therapists must balance health protection, patient autonomy, and equity in access to therapy.

#### Communication and Quality of Relationship

The second group of concerns related to communication and the quality of relationships in the need to cover the face with masks. During the pandemic, psychotherapists encountered ethical dilemmas related to the need to maintain spatial distance and wear masks, which affect the quality of communication and therapeutic relationships. These limitations can create tensions between the values of health safety and the desire to establish the best possible therapeutic relationship based on empathy, trust, and deep contact. Research clearly indicates the important role of nonverbal communication in the therapeutic relationship, where the behaviors and nonverbal signals of both the therapist and patient play a key role in building a therapeutic alliance and providing information about the emotions experienced [56,67]. The expression and perception of emotions that we have access to through nonverbal communication are particularly important in the context of psychotherapy [68]. Wearing masks, while necessary for health protection, proves to be frustrating and uncomfortable at the same time, making it difficult to communicate freely [69]. Masks also interfere with the ability to fully read emotions from facial expressions, which has been confirmed by research indicating difficulties in the perception of the emotions that masks cause [70-72]. Therefore, a dilemma may arise between safety and the quality of the therapeutic relationship, as well as between emotional transparency and public health.

It seems that what may be helpful in these dilemmas is to conduct psychotherapy in a hybrid model, combining in-person and remote meetings, which would give a chance to minimize the discomfort associated with masks and distance. In addition, openly discussing with the patient how masks and distance affect the therapeutic relationship can help the patient to feel better understood and involved in the therapeutic process.

#### Technical and Logistical Limitations

The third group of ethical dilemmas related to conducting face-to-face sessions during the pandemic was related to technical and logistical aspects, which included the need to ensure appropriate hygienic conditions, such as disinfecting surfaces, following sanitary guidelines, and enforcing these rules among patients, as prevention, according to experts, was the main strategy to control and prevent the spread of the virus. This gave rise to a dilemma among psychotherapists between health safety and the comfort and efficiency of work and between the responsibility for compliance with the guidelines by patients and the autonomy of these patients. Therapists had to balance their responsibilities in maintaining office hygiene and health protection, which involved additional stress. On the one hand, they felt obliged to provide safe working conditions for patients and themselves; on the other hand, sanitary guidelines could limit the time and resources allocated to therapeutic work.

In addition, psychotherapists faced the need to enforce compliance with patient restrictions, which could create tension in the therapeutic relationship. In a situation where the patient was reluctant to follow the recommendations (eg, wearing a mask), the therapist had to decide between the need to ensure safety and respect for the patient’s autonomy, which could have a negative impact on the therapeutic process.

According to psychotherapists, these are time-consuming and costly challenges, which put an additional burden on them, both financially and emotionally. In a situation where psychotherapists had to meet increasing sanitary requirements while striving to provide high-quality psychotherapeutic services, tension arose between the values of health safety and the efficiency and comfort of work.

Perhaps it would be helpful for therapists in this situation to introduce clear guidelines on sanitary rules so that everyone is aware of the applicable rules before starting psychotherapy. It seems that this is a broader problem than just the pandemic period, as similar dilemmas can accompany psychotherapists in the event of patients who are sick or have a cold visiting offices for sessions.

### Limitations and Further Research

This study has several limitations. First, our research was conducted among a voluntary group of therapists from 4 European countries, which does not reflect the entire professional population. The sample is not representative, as the study is dominated by women and people practicing in large cities, and private practices. Future studies should include underrepresented groups. It should also be noted that despite the use of multi-channel recruitment and comparison of early and late responder profiles, participation in the survey required independent initiative, which may be associated with a potential selection bias, and limit the possibility of full generalization of results. It is, therefore, not possible to generalize the obtained results to the entire population of professional psychotherapists.

Second, it should also be noted that different countries have been in different phases of the pandemic and some therapeutic societies have already issued recommendations for online therapy, while others have not yet done so. Therefore, it can be expected that studies conducted at a different time of the pandemic and imposed restrictions could yield different results. Growing experience in online work itself may be an element that reduces certain dilemmas or concerns. It seems important to further explore the analyzed topic of ethical dilemmas in online and face-to-face work, because the topic does not lose its relevance. On the one hand, online work is becoming better known and more common; on the other hand, the period of restrictions and lockdowns has come to an end. The context of ethical dilemmas in the work of psychotherapists does not lose its relevance, and it seems important to continue the analyzed thread in scientific research.

It is worth continuing the research, covering a larger number of countries and diverse groups of respondents, which could help identify new dilemmas and check the extent to which some difficulties have been solved in the postpandemic period. It also seems reasonable to expand the research to include variables related to the patient’s perspective to better understand how they perceive online and in-person therapy in the context of ethical challenges reported by therapists.

This type of research, along with the development of clear ethical and legal guidelines, can support psychotherapists in providing safe and effective therapy both online and in an in-person form. In the context of the pandemic itself, research by Pierce et al [73] shows its potential negative impact on mental health, suggesting an increased need for psychotherapy.

### Conclusions

On the basis of the identified ethical dilemmas and the principles of Beauchamp and Childress [74], we recommend the following:

1. A hybrid therapy model based on the development of flexible guidelines that allow therapists to combine online and in-person sessions according to the clinical needs and preferences of the patient, while considering risk factors (eg, immunologic status) and the principle of justice, ensuring fair access to services.
2. A crisis protocol, that is, the creation of a standardized decision-making algorithm to support therapists in choosing the appropriate form of intervention (online vs face-to-face), prioritizing health protection (beneficence and nonmaleficence), while also respecting patient autonomy.
3. Digital competence training, that is, the introduction of targeted training programs that address both technical aspects (eg, data security and telemedicine tools) and interpersonal skills essential for establishing a therapeutic relationship in a online setting. This will enable therapists to conduct online therapy in an ethical and effective manner.

The implementation of these recommendations should involve ongoing dialogue with representatives of psychotherapy associations and be adapted to local systemic and legal contexts.

## Abbreviations

AI: artificial intelligence
COREQ: Consolidated Criteria for Reporting Qualitative Studies

## Data Availability

The data that support the findings of this study are not publicly available because they may contain information that may violate the privacy of research participants. In addition, the results presented are part of a larger project whose data will be used later.
